# Attitude and Perception Toward Taking Selfies and Using Filters and Their Relationship With Blepharoplasty

**DOI:** 10.7759/cureus.30426

**Published:** 2022-10-18

**Authors:** Saif K Dossari, Ahmed Z Alkhars, Mahdee Albahrani, Mohammed H Alam, Ahmed Alali, Sarah K Aljamri, Hussam F Alkhars

**Affiliations:** 1 Surgery, King Faisal University, Hofuf, SAU; 2 General Physician, AlJaber Eye and ENT Hospital, Al-Ahsa, SAU; 3 General Physician, AlJaber Eye and ENT Hospital, Hofuf, SAU; 4 General Physician, AlJaber Eye and ENT Hospital, Khobar, SAU; 5 Medicine, King Fahad University Hospital, Dammam, SAU; 6 Medicine and Surgery, King Faisal University, Alhofuf, SAU

**Keywords:** cosmetic surgeries, using photo filters, taking selfies, social media, blepharoplasty

## Abstract

Background: Due to the general increase in the use of social media, the increasing popularity of taking selfies and using filters, we found it essential to examine the effect of these behaviors on the perception and attitude toward blepharoplasty.

Aim: This article was conducted to assess participants’ attitudes and perceptions toward taking selfies and using filters and their relation to blepharoplasty.

Methods: This study was an observational cross-sectional study undertaken in Saudi Arabia. The study targeted all adults in Saudi Arabia. The study subjects are adults living in Saudi Arabia who consented to participate in the study and have filled out the questionnaire fully between January and April 2022 while meeting the inclusion and exclusion criteria. A convenient sampling technique was used for data collection. The Chi-square test was used to test for association.

Results: A total of 466 participants were included in the study. (94.6%) of the participants reported taking selfies, with varying frequencies, with Snapchat being the most commonly used application (82.5%). Moreover, 87.05% of the participants reported using filters, and 96.08% of those who use filters used them from Snapchat. 45.5% of the participants reported comparing their eyelids with others' when seeing their selfies, 50.6% reported thinking that taking a selfie has a role in making a decision to undergo blepharoplasty, and 47.6% reported thinking that using filters has a role in making a decision to undergo blepharoplasty.

Conclusion: This study reflected a notably high rate of taking selfies and using photo filters. The participants' assessment toward the impact of taking selfies and using filters on the decision to undergo blepharoplasty was observed to be moderate. Females were observed to have significantly higher rates of thinking that taking pictures and using filters influence the decision to undergo blepharoplasty compared to males.

## Introduction

In recent years with the emergence of social media and the era of social networking programs, people tend to idealize their profiles, and one method of this idealization is to share the perfect picture, and in order to share that perfect picture, people often use filters to improve their profile appearance [1].

Social media platforms have become a very important medium for sharing ideal body images with others, and this is shown to have a negative effect on the self-image of users. Research showed that the use of social media platforms was related to an increase in the willingness of users to undergo an aesthetic surgical procedure [2,3].

A selfie, which is a very popular activity on social media platforms, is a photograph or an image that has been taken by oneself, usually using a cell phone, and shared with others, recent research found that the use of selfies rather than the general use of social media was more predictive of self-image dissatisfaction, which is a known risk factor for considering cosmetic surgery [4-6].

Selfies allow users to self-check their pictures and edit them to improve them until it reaches the ideal picture of themselves. This process leads to self-dissatisfaction, since users will notice the difference between the edited image and their actual image, and this might promote the desire to improve their appearance by considering cosmetic surgery [1].

The rate of aesthetic surgeries has increased generally, in fact, in 2021 aesthetic surgical procedures showed a 54% increase in comparison to the previous year. Blepharoplasty is a surgical procedure that addresses the aesthetic appearance and the function of the upper and lower eyelids, it is done for eyelids defects and deformities correction, and cosmetic appearance improvement [7]. Blepharoplasty was the sixth most common cosmetic procedure with 149,668 surgeries done in 2021, 72% more than the previous year [8].

Due to the general increase in the use of social media, and the increasing popularity of taking selfies and using filters, we found it essential to examine the effect of these behaviors on the perception and attitude toward blepharoplasty. This article was conducted to assess participants’ attitudes and perceptions toward taking selfies and using filters and their relation to blepharoplasty. As exploring this issue would generate a better understanding of the unexpected influencing factors toward blepharoplasty.

## Materials and methods

Study design and settings

This study was an observational cross-sectional study undertaken in Saudi Arabia. The study targeted all adults in Saudi Arabia.

Study subjects, inclusion, and exclusion criteria 

The study subjects are adults living in Saudi Arabia who consented to participate in the study and have filled out the questionnaire fully between January and April 2022 while meeting the inclusion and exclusion criteria. 

The inclusion criteria were being 18 years and older, consenting to participate in the study, and living in Saudi Arabia. The exclusion criteria were those who were younger than 18 years old, those with no consent to participate, not living in Saudi Arabia, and participants with missing data.

Sample size and sampling

Convenient random sampling was used for the collection of data. An online survey was spread on social media with an invitation to fill it up. The minimum required sample size was concluded using the formula n = z2pq\d 2. With a confidence level of 95%, an estimated proportion of 50%, a 5% level of precision, and an estimated population of 34 million (the latest Saudi population). The minimum sample size was calculated to be 385; however, a higher number was included in the study to ensure higher accuracy.

Study tool and its validation

For the sake of validating the used study tool, a questionnaire was established by the authors and was presented to three consultants in ophthalmology. The questionnaire was revised by them, modified, and then they affirmed using it. The survey was first made in English, then it was presented to a language expert which has approved it after minor grammatical and linguistic editing, then the linguist translated it into Arabic. It was then translated back to English by another linguist to ensure accurate translation, minor changes were done in the Arabic version to achieve a more appropriate translation. Lastly, a pilot study was conducted on a small group of participants (28 persons) to ensure a uniform understanding of the questionnaire content.

Data collection

An online survey was established in Google forms for collecting data. The online survey was spread on social media platforms and invited adults living in Saudi Arabia to participation. Data were collected through a self-administered fashion where participants first consented to participate in the study before starting to fill out the survey. The questionnaire included four sections, the first section asked about sociodemographic information, the second section asked about medical history, the third section asked about previous plastic surgery, and the fourth and last section asked about participants' practices and perception toward taking selfies and the influence of taking selfies on the perception toward blepharoplasty.

Data management and statistical analysis

Data were collected in the benign in google form, then it was downloaded into a Microsoft Excel sheet. After that, data was transferred for analysis in Statistical Package for the Social Sciences, SPSS 23rd version (IBM Corp., Armonk, NY). Frequency and percentages were used to display categorical variables. Chi-square tests were used to test for association. The level of significance was set at 0.05.

Confidentiality and ethical consideration

Data were managed with utmost confidentiality; privacy was maintained throughout all study steps. Ethical approval was obtained from the ethical board of King Faisal University (reference code: KFU-REC-2021- NOV-EA000102).

## Results

A total of 466 participants was included in this study. Table 1 shows the sociodemographic profile of the participants. 101 (21.7%) of the participants were males, while 365 (78.3%) were females. As for the age, the mean was 34.12 +8.47. 

**Table 1:** Socio-demographic profile of the participants (n = 466)

Demographical Characteristics	n	%
Gender		
Male	101	21.70
Female	365	78.30
Age		
Mean	34.12	
Standard deviation	8.47	

**Figure 1:**
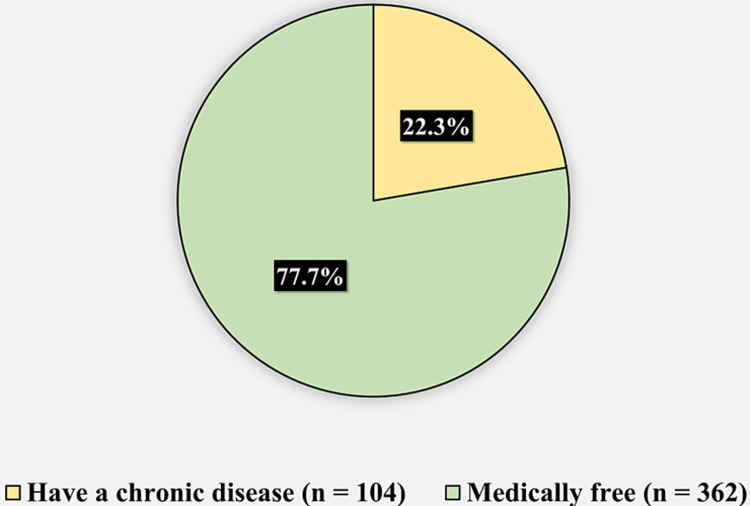
Medical history of the participants

Figure 1 displays the medical history of the participants. One hundred four (22.3%) of the participants had a chronic disease, while 362 (77.7%) of the participants were medically free.

**Figure 2:**
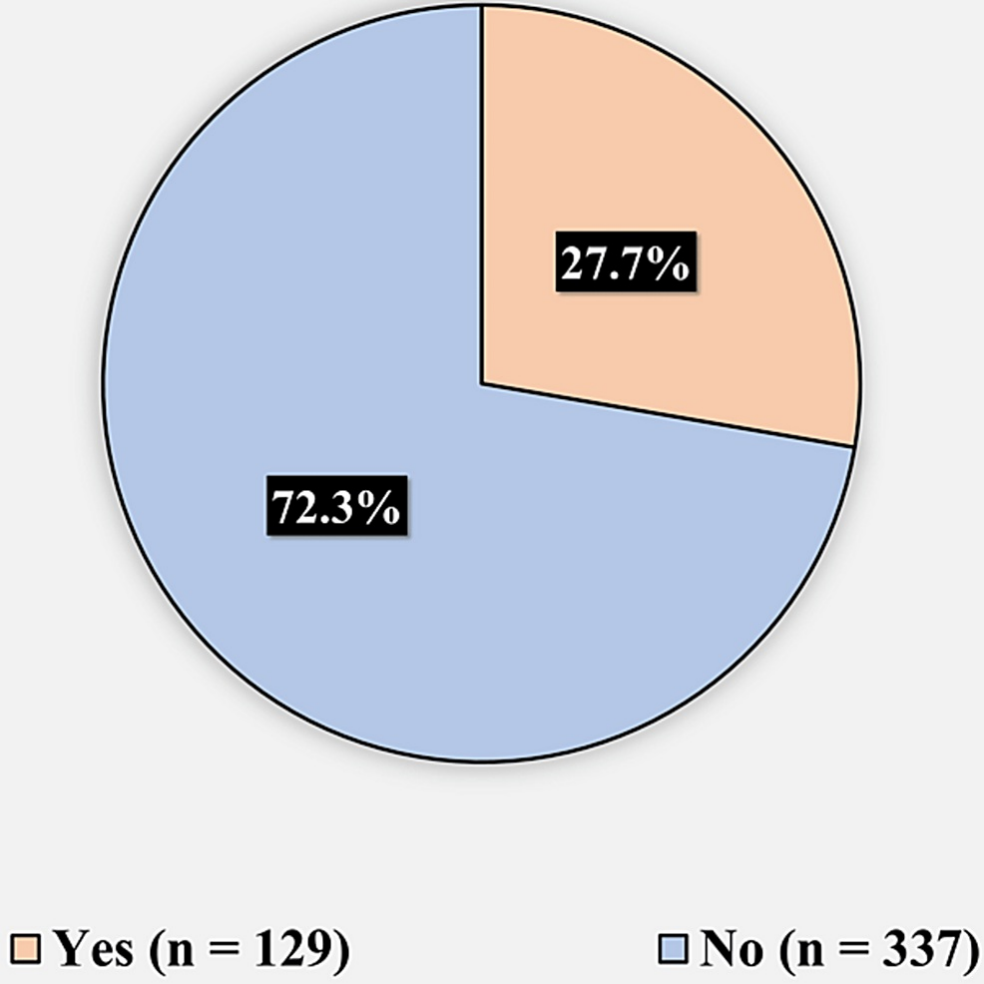
History of undergoing plastic surgery (in face, nose, eyelids, cheeks, or chin)

Figure 2 presents the history of undergoing plastic surgery (in the face, nose, eyelids, cheeks, or chin). One hundred twenty-nine (27.7%) reported undergoing a plastic surgery from before, while 337 (72.3%) reported never undergoing plastic surgery before. 

**Figure 3:**
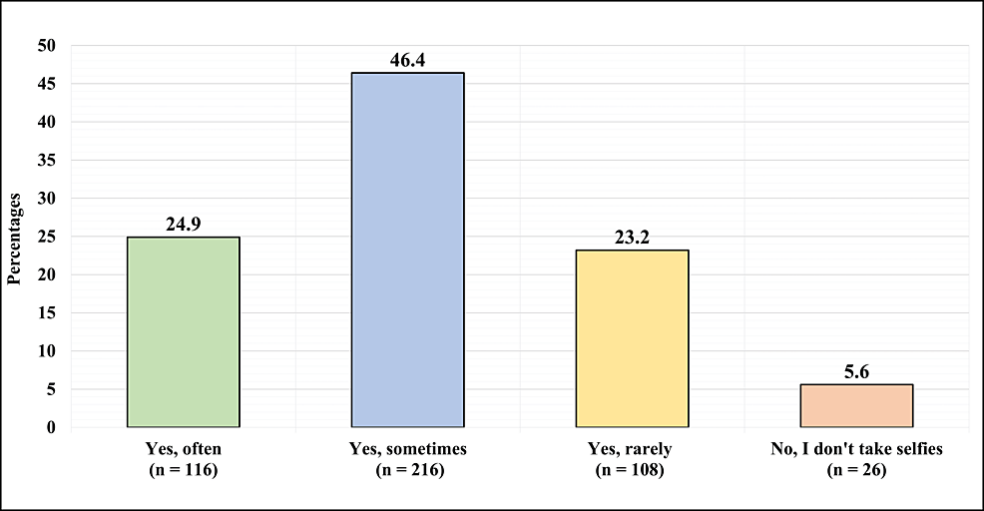
Do you take selfies?

Figure 3 demonstrates the participants responses toward “do you take selfies?” Twenty-six (5.6%) reported they don’t take selfies at all, while 440 (94.42%) reported they take selfies. One hundred sixteen (24.9%) reported they take selfies often, 216 (46.4%) reported they take selfies sometimes, and 108 (23.2%) reported they take selfies rarely.

**Figure 4:**
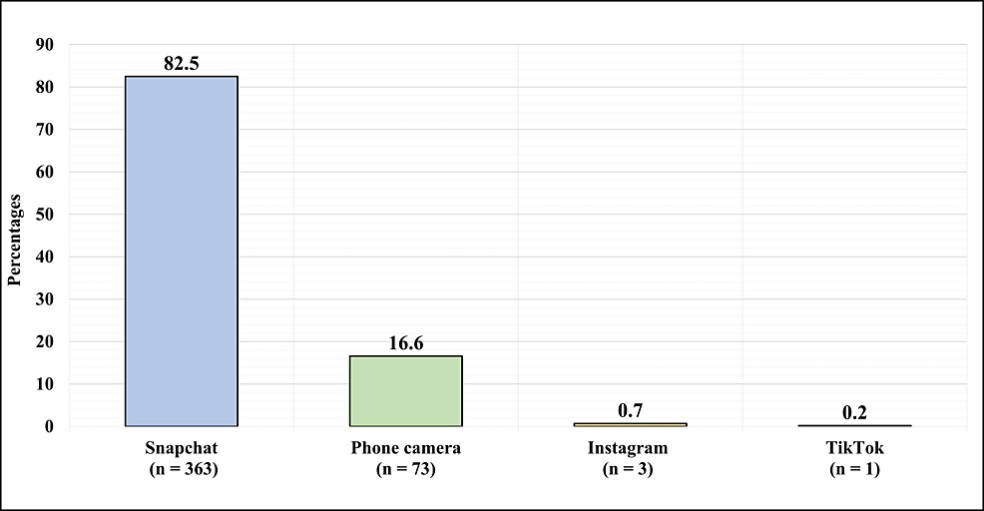
What is the application you use the most for taking selfies?

Figure 4 illustrates the applications the participants use the most for taking selfies. The most commonly used application was Snapchat 363 (82.5%), followed by phone camera 73 (16.6%), followed by Instagram three (0.7%), and lastly TikTok one (0.2%).

Table 2 shows the participants attitude of taking selfies and using filters. Multiple questions were directed only for those 440 (94.42%) who reported taking selfies. As for how often they take selfies, 288 (65.45%) reported raking it on occasions and special moments, 102 (23.18%) reported taking it once every few days, 38 (8.64%) reported taking it one to five times daily, five (1.14%) reported using it six to 10 times daily, and seven (1.59%) reported using it more than 10 times a day. As for using filters when taking selfies, 57 (12.95%) reported not using filters at all, while 383 (87.05%) reported using filters. 188 (42.73%) reported always using filters, and 195 (44.32%) reported using filters sometimes. As for the applications the participants use the most for applying filters, the most commonly used application was Snapchat by 368 (96.08%), Instagram by six (1.57%), and other applications by nine (2.35%). Among those who use filters, 329 (85.9%) reported they share their selfies, while 54 (14.1%) reported they do not share their selfies. When the participants were asked if they share their unfiltered selfies, 203 (46.14%) reported they do share their unfiltered selfies, while 237 (53.86%) reported they do not share unfiltered selfies with others. All the participants were asked if they compare their eyelids to others when they see their selfies, 212 (45.5%) said yes, they do, while 254 (54.5%) said they do not.

**Table 2:** Participants attitude of taking selfies and using filters

Question	n	%
Questions Directed for Those who Take Selfies		
How often do you take selfies? (n = 440)		
On occasions and special moments	288	65.45
Once every few days	102	23.18
1-5 times a day	38	8.64
6-10 times a day	5	1.14
More than 10 times a day	7	1.59
Do you use filters when taking a selfie? (n = 440)		
Yes, sometimes	195	44.32
Yes, always	188	42.73
I don't use filters	57	12.95
If you use filters on your selfies, what application do you use the most for applying filters on pictures? (n = 383)		
Snapchat	368	96.08
Instagram	6	1.57
Other applications	9	2.35
For those who uses filters on their selfies, do you share filtered selfies with others? (n = 383)		
Yes	329	85.90
No	54	14.10
Do you share unfiltered selfies with others? (n = 440)		
Yes	203	46.14
No	237	53.86
Questions Directed for All Participants (n = 466)		
Do you compare your eyelids with others' when you see their selfies?		
Yes	212	45.50
No	254	54.50

Table 3 presents the participants perception toward the effect of taking selfies and using filters on the decision to undergo blepharoplasty. 236 (50.6%) of the participants thought that taking a selfie has a role on making a decision to do an eyelids plastic surgery, while 230 (49.4%) thought it did not. Two hundred twenty-two (47.6%) of the participants thought that using a filter has a role on making a decision to do an eyelids plastic surgery, while 244 (52.4%) thought it did not.

**Table 3:** Participants perception toward the effect of taking selfies and using filters on the decision to undergo blepharoplasty (n = 466)

Question	n	%
Do you think taking a selfie has a role on making a decision to undergo blepharoplasty?		
Yes	236	50.60
No	230	49.40
Do you think using a filter has a role on making a decision to undergo blepharoplasty?		
Yes	222	47.60
No	244	52.40

Table 4 displays the factors associated with participants thought toward the influence of taking selfies on the decision to undergo blepharoplasty. Gender was significantly associated with the participants thought toward the influence of taking selfies on the decision to undergo blepharoplasty (p = 0.04), where it was observed that females had a significantly higher rate of thinking that taking selfies had a role in the decision to undergo blepharoplasty compared to males (53.2% vs 41.6%). Age, medical history, history of undergoing plastic surgery, behavior of taking selfies, behavior of using filters were all not significantly associated with the participants thought toward the influence of taking selfies on the decision to undergo blepharoplasty.

**Table 4:** Factors associated with participants thought toward the influence of taking selfies on the decision to undergo blepharoplasty *Significant at level 0.05

Factor	Do you think taking a selfie has a role on making a decision to undergo blepharoplasty?		P-value
	Yes	No	
Gender (n, %)			0.04*
Male	42 (41.6%)	59 (58.4%)	
Female	194 (53.2%)	171 (46.8%)	
Medical History (n, %)			0.207
Have a chronic disease	47 (45.2%)	57 (54.8%)	
Medically free	189 (52.2%)	173 (47.8%)	
History of Undergoing Plastic Surgery (n, %)			0.334
Yes	70 (54.3%)	59 (45.7%)	
No	166 (49.3%)	171 (50.7%)	
Do you take selfies? (n, %)			0.201
Yes	226 (51.4%)	214 (48.6%)	
No	10 (38.5%)	16 (61.5%)	
Do you use filters when taking a selfie? (n, %)			0.705
Yes	185 (48.3%)	198 (51.7%)	
No	26 (45.6%)	31 (54.4%)	
Age (mean +/- standard deviation)	33.82 +/- 8.12	34.42 +/- 8.82	0.455

Table 5 demonstrates the factors associated with participants thought toward the influence of using filters on the decision to undergo blepharoplasty. Gender was significantly associated with the participants thought toward the influence of using filters on the decision to undergo blepharoplasty (p = 0.006), where it was observed that females had a significantly higher rate of thinking that using filters had a role in the decision to undergo blepharoplasty compared to males (51% vs 35.6%). Age, medical history, history of undergoing plastic surgery, behavior of taking selfies, behavior of using filters were all not significantly associated with the participants thought toward the influence of using filters on the decision to undergo blepharoplasty.

**Table 5:** Factors associated with participants thought toward the influence of using filters on the decision to undergo blepharoplasty *Significant at level 0.05

Factor	Do you think using a filter has a role on making a decision to do undergo blepharoplasty?		P-value
	Yes	No	
Gender (n, %)			0.006*
Male	36 (35.6%)	65 (64.4%)	
Female	186 (51%)	179 (49%)	
Medical History (n, %)			0.571
Have a chronic disease	47 (45.2%)	57 (54.8%)	
Medically free	175 (48.3%)	187 (51.7%)	
History of Undergoing Plastic Surgery (n, %)			0.346
Yes	66 (51.2%)	63 (48.8%)	
No	156 (46.3%)	181 (53.7%)	
Do you take selfies? (n, %)			0.575
Yes	211 (48%)	229 (52%)	
No	11 (42.3%)	15 (57.7%)	
Do you use filters when taking a selfie? (n, %)			0.705
Yes	185 (48.3%)	198 (51.7%)	
No	26 (45.6%)	31 (54.4%)	
Age (mean +/- standard deviation)	33.96 +/- 7.84	34.26 +/- 9.02	0.700

## Discussion

The use of social media platforms has been extensive in the last decade, especially by young adults. There are many applications that are programmed to enhance the appearance of pictures taken by users, and it also been used extensively to improve the users’ body image to viewers of their profiles, and this might affect users’ self-image negatively, driving users to seek cosmetic surgery to enhance that self-image and look like their “filtered” profile picture. Hence, the use of filters might be a motivator for users to seek undergoing blepharoplasty, and behind this hypothesis, the research idea was formulated. In addition to that, the International Society of Aesthetic Plastic Surgery reported in a study that Saudi Arabia ranks 29 among the top 30 countries with the highest performed cosmetic surgeries, especially eyelid aesthetic surgeries [9]. To our knowledge, this study is the first to measure the association between taking selfies and using filters and the attitude and perception toward blepharoplasty. As the work regarding the influence of social media, taking selfies, and using filters on the perception and attitude toward blepharoplasty is scarce, the discussion will be comparing the results of all areas of cosmetic surgeries rather than only blepharoplasty.

Multiple studies have noted that social media have a negative effect on the self-image and self-esteem of users as it exposes the users to unrealistic beauty expectations and frequent comparisons to other users. In a study about the effect of women's mood and body image upon viewing images of attractive celebrities, the results showed that it had a negative effect on mood and body image [10]. In another study on the effect of social media and the factors affecting the body image of users, the results were consistent with the previously mentioned study, showing a negative effect on mood and body image [11]. Social media does not only negatively impact self-image and mood, but a study has also proven that social media contribute to and influence appearance-changing attitudes [12]. In this study, 212 (45.5%) reported comparing their eyelids with others when seeing their selfies, 236 (50.6%) thought taking a selfie has a role in making a decision to undergo blepharoplasty, and 222 (47.6%) thought using a filter has a role on making a decision to undergo blepharoplasty, which is consistent with the finding from the previously mentioned studies, where participants agreed that there is a direct relation between taking selfies, using filters, and the perception toward undergoing blepharoplasty. Moreover, Arab et al. in their research reported that those who used social media for more than five hours per day were among the participants who were highly influenced by advertisements for cosmetic surgeries [13]. Arab et al. have further enumerated that the underlying motive behind seeking aesthetic consultation among women was the pressure they experienced from social media and peers rather than their desire to change their appearance. Ashikali et al. reported similar findings to Arab et al., where they stated that making advertisements for cosmetic surgeries contributed toward both self-discrepancy and deciding to undergo aesthetic surgeries [14]. Likewise, Walker et al. showed in their work that their female participants who used social media at high frequencies had a significantly higher tendency to desire cosmetic surgeries [12]. Other factors that enhance cosmetic surgeries seeking behaviors were reported by Furnham et al., which included low self‑esteem, decreased life satisfaction, low self‑rated physical attractiveness, and weak religious beliefs [15].

In this study, the most commonly used application for taking selfies and using filters was Snapchat (82.5%) and (96.08%) respectively. Alghamdi et al. in their work reported that social media applications in general and Snapchat in specific had a huge impact on the decision to undergo rhinoplasty [16]. On the other hand, Alghonaim et al. [17], Park et al. [18], and AlAlshaalan et al. [19] stated that the platform with the greatest influence on the perception and decision to undergo aesthetic procedures was Instagram. Aldosari et al. [20] in their work reported they the majority of patients seeking consultation in plastic surgery clinics were impacted by the before and after photos, they encountered on social media. Similarly, AlAlshaalan et al. stated in their work that 91.6% of the participants reported being influenced in their decision to undertake oculoplastic surgeries by seeing before and after operation photos.

In this study, it was found that females had a significantly higher rate of believing that taking selfies and using filters respectively influence the decision to undergo blepharoplasty. Likewise, Henderson‑King et al. [21] found that considering undergoing cosmetic surgeries was more prevalent in females, as they are more concerned and caring toward their physical appearance.

Strengths and limitations

This study had multiple limitations such as the nature of the study and the targeted population. This study was a cross-sectional study that targets the general population, ideally, it would have been more precise and accurate to conduct a retrospective study that targets the patients who underwent blepharoplasty and assess the influence of taking selfies, using filters, and the use of social media on the perception and attitude toward undergoing blepharoplasty. However, access to the patients who underwent blepharoplasty was not feasible, and conducting this study allowed us to take a glimpse at the overall situation of this topic among the general population. A study with a greater sample size would generate a more precise view and findings on this topic. Another limitation was that this study only targeted the population's opinion toward blepharoplasty, exploring the influence of social media use and taking selfies on overall facial plastic surgeries would be more wholistic.

The strength of this study lies in spotting the light on a subject not well explored. This study widens the view over the topic and allows opening more opportunities to further investigating the influence of the new rising trend of social media use, taking selfies, and using filters on the perception and attitude toward blepharoplasty in specific and facial plastic surgeries in general.

Recommendations

We recommend conducting retrospective nationwide studies that target patients who underwent blepharoplasty and assess the impact of social media, taking selfies, and using filter behavior on the perception and attitude toward blepharoplasty and facial plastic surgeries in general. We also recommend conducting community campaigns the rise awareness of the possible negative impact of social media use on patients' self-satisfaction toward their appearance and to further enhance embracing self-beauty and rise confidence.

## Conclusions

This study reflected a notably high rate of selfie and filter use, where (94.6%) of the participants reported taking selfies, with varying frequencies, with Snapchat being the most commonly used application (82.5%). Moreover, 87.05% of the participants reported using filters, and 96.08% of those who use filters used them from Snapchat. Moreover, the participants reflected a perception that suggests a moderate influence of using selfies and filters on the decision to undergo blepharoplasty, where 45.5% of the participants reported comparing their eyelids with others' when seeing their selfies, (50.6%) reported thinking that taking a selfie has a role in making a decision to undergo blepharoplasty, and 47.6% reported thinking that using filters has a role in making a decision to undergo blepharoplasty.

## References

[1] Sun Q (2021). Selfie editing and consideration of cosmetic surgery among young Chinese women: the role of self-objectification and facial dissatisfaction. Sex Roles.

[2] Holland G, Tiggemann M (2016). A systematic review of the impact of the use of social networking sites on body image and disordered eating outcomes. Body Image.

[3] de Vries D, Peter J, Nikken P, de Graaf H (2014). The effect of social network site use on appearance investment and desire for cosmetic surgery among adolescent boys and girls. Sex Roles.

[4] Chua T, Chang L (2016). Follow me and like my beautiful selfies: Singapore teenage girls’ engagement in self-presentation and peer comparison on social media. Computers Human Behavior.

[5] Cohen R, Newton-John T, Slater A (2018). ‘Selfie’-objectification: the role of selfies in self-objectification and disordered eating in young women. Computers Human Behavior.

[6] Niu G, Sun L, Liu Q, Chai H, Sun X, Zhou Z (2019). Selfie-posting and young adult women’s restrained eating: the role of commentary on appearance and self-objectification. Sex Roles.

[7] Carraway JH, Tran P (2009). Blepharoplasty with ptosis repair. Aesthet Surg J.

[8] (2022). Aesthetic plastic surgery national databank STATISTICS 2020-2021. https://cdn.theaestheticsociety.org/media/statistics/2021-TheAestheticSocietyStatistics.pdf.

[9] Reham A (2019). The reasons behind the trending of facial plastic surgery in Saudi Arabia. Scholarly J Otolaryngol.

[10] Brown Z, Tiggemann M (2016). Attractive celebrity and peer images on Instagram: effect on women's mood and body image. Body Image.

[11] Fardouly J, Pinkus RT, Vartanian LR (2017). The impact of appearance comparisons made through social media, traditional media, and in person in women's everyday lives. Body Image.

[12] Walker C, Krumhuber E, Dayan S, Furnham A (2019). Effects of social media use on desire for cosmetic surgery among young women. Current Psychol.

[13] Arab K, Barasain O, Altaweel A (2019). Influence of social media on the decision to undergo a cosmetic procedure. Plast Reconstr Surg Glob Open.

[14] Ashikali E, Dittmar H, Ayers S (2017). The impact of cosmetic surgery advertising on Swiss women’s body image and attitudes toward cosmetic surgery. Swiss J Psychol.

[15] Furnham A, Levitas J (2012). Factors that motivate people to undergo cosmetic surgery. Plastic Surgery.

[16] Alghamdi W, Qobty A (2020). Effect of social media on decision to undergo rhinoplasty. Global J Otolaryngol.

[17] Alghonaim Y, Arafat A, Aldeghaither S, Alsugheir S, Aldekhayel S (2019). Social media impact on aesthetic procedures among females in Riyadh, Saudi Arabia. Cureus.

[18] Park SS, Akella SS, Moon JY, Zarrin B, Patel S, Doshi H, Barmettler A (2020). Building your brand: Analysis of successful oculoplastic surgeons on social media. Ophthalmic Plast Reconstr Surg.

[19] Alshaalan H, AlTamimi L, Alshayie R, Alsuhaibani A (2021). The impact of social media accounts on periocular cosmetic surgeries. Saudi J Ophthalmol.

[20] Aldosari B, Alkarzae M, Almuhaya R, Aldhahri R, Alrashid H (2019). Effect of media on facial plastic surgery in Saudi Arabia. Cureus.

[21] Henderson-King D, Henderson-King E (2005). Acceptance of cosmetic surgery: scale development and validation. Body Image.

